# Epidemiological analysis and risk prediction of scrub typhus from 2006 to 2021 in Sichuan, China

**DOI:** 10.3389/fpubh.2023.1177578

**Published:** 2023-05-30

**Authors:** Yao Zhang, Mengyuan Zhang, Yao Qin, Lun Zhang, Dianju Kang, Rongjie Wei, Changhong Yang

**Affiliations:** Department of Emergency Management, Sichuan Center for Diseases Control and Prevention, Chengdu, China

**Keywords:** scrub typhus (tsutsugamushi disease), BRT model, epidemiological feature, contributing variables, risk areas

## Abstract

**Background:**

In the past decade, the number of reported cases of scrub typhus (ST) has increased dramatically in Sichuan Province. We aimed to overview the epidemiological characteristics of ST, identify the variables contributing to the spatial distribution, and estimate the risk areas of ST occurrence.

**Methods:**

Daily ST cases reported at the county level from 2006 to 2021 and datasets on environmental and socioeconomic variables were obtained. Joinpoint regression model was utilized to examine the incidence trends and to calculate the annual percentage change. Global spatial autocorrelation analysis was employed to explore the spatial temporal patterns. Then BRT model was employed to identify variables that make sense and predict the risk areas of ST occurrence.

**Result:**

It has been reported that there were 6,338 ST cases in Sichuan Province from 2006 to 2021, and the incidence rates continued to rise. Most cases were distributed between June and October each year, peaking in August. During the study period, the cases showed spatial clustering at the county level, mainly in the Panxi area, and then slowly spread to the northwest and northeast. Shrubs, precipitation, farmland and maximum temperature were the primary variables that affected the spatial distribution of this disease. It was estimated that the areas including Liangshan, Panzhihua, Bazhong, and Guangyuan were most at risk of transmission. and there were approximately 32.315 million people living in the areas with potential risk of infection throughout Sichuan.

**Conclusion:**

Many counties in Sichuan Province were estimated to be susceptible to ST. Our found in this data-driven study could be used to guide the implementation of targeted prevention and control measures in high-risk areas.

## 1. Introduction

Scrub typhus (ST) is a vector-borne disease transmitted through the bites of chigger mites carrying *Orientia tsutsugamushi* (1–3). The primary manifestations of the disease are eschar, fever, headache, systemic lymphadenopathy, rash, etc. (4–6). Delayed treatment may result in pneumonia, myocarditis and even death (7, 8). Generally, chiggers are only active within a certain area of their breeding site. However, alterations in human behavior and social environment lead to migration of vectors and hosts from one location to another, which may spread pathogens to more distant areas and cause disease transmission (9–11). ST is widespread in the “tsutsugamushi triangle” area ranging from Afghanistan in the west to the east coast of Russia in the north to northern Australia in the south (12). Approximately 1 billion people worldwide are at risk of infection, leading to at least 1 million ST cases each years (13, 14). Recently, the incidence rates of ST have begun to rise in known endemic areas such as India and Korea (15, 16), while cases have been widely reported in tropical and subtropical regions far from the “tsutsugamushi triangle” (17–19), which may be an indication for the resuscitation of this unheeded disease (13, 20).

In China, since the first ST case was detected in southern Guangdong in 1948, the natural focus of ST has been limited to the area south of the Yangtze River (21, 22). Until the 1980s, the endemic area began to expand slowly northward and westward, and multiple local outbreaks occurred (23, 24). Currently, ST cases have spread across China, and cases have been discovered in both urban and rural areas in all provinces (25). Recently, the incidence rates of the disease in our country have risen rapidly with the acceleration of urbanization, tourism exploitation of natural environment, climate alteration, and migration movement, which has become an important health problem (26–28). Sichuan Province is the focus of ST in southern China, faced with the dilemma of rapid increase in the incidence of ST (29). However, research on ST in this area is still limited.

Based on available genomic and genotyping studies (30), the antigenic and genetic diversity of *Orientia tsutsugamushi* has become a primary impediment to the development of effective vaccines, making the prediction of risk regions even more important at this stage. In recent years, spatial analysis and ecological modeling have been utilized to investigate the influencing factors associated with ST and to predict epidemic risk (28, 31). Previous studies have successfully estimated the distribution of ST in southern China (32), but studies on the distribution of the disease in Sichuan Province have still not been conducted. In this research, we analyzed the epidemiological characteristics of ST in Sichuan Province from 2006 to 2021. Transmission patterns of the disease were modeled to identify environmental and socioeconomic variables affecting distribution, and to forecast the areas at risk in Sichuan Province.

## 2. Materials and methods

### 2.1. Study area

Sichuan Province is located in southwest China, having jurisdiction over 21 prefectures and 183 counties. By the end of 2020, the resident population was 83.71 million. Sichuan has complex landforms, rich soil types and obvious regional climate differences. It could be categorized into three major climatic zones in accordance with the differences in water, heat and light conditions. Briefly, the basin is a subtropical humid climate zone, warm and humid throughout the year; the southwest mountainous area is a subtropical and semi-humid climate zone, with a higher average annual temperature; and the northwest plateau is an alpine climate zone, with large altitude differences and obvious climate alterations (33).

### 2.2. Data collection and management

All the data of scrub typhus cases from January 2006 to December 2021 were obtained from China’s National Statutory Infections Disease Reporting Information System. The data covered all reported probable cases (clinically diagnosed) and confirmed cases (laboratory diagnosed), incorporating information including gender, year, occupation, time of onset, time of reporting, and place of residence. Case definition was based on the guidelines issued by the Chinese Center for Disease Control and Prevention^1^ (details was displayed in Supplementary material). Probable cases were diagnosed by physicians based on epidemiological exposure (field activities in endemic areas 1–3 weeks before illness onset) and clinical manifestations (fever, specific eschar, or ulcers). A confirmed case was defined as a probable case meeting any of the following criteria: (1) a positive Weill-Felix test; (2) a positive indirect immunofluorescence antibody assay; (3) positive PCR; (4) the pathogen was isolated (34).

All the cases data in the current research were anonymized. And the cases data were not subject to institutional review board assessment.

Environmental variables were collected to investigate the potential contributing variables on the spatial distribution of ST. We extracted the monthly cumulative sunshine hours, monthly cumulative precipitation, monthly maximum temperature, and monthly minimum temperature between January 2006 and December 2020 from the National Earth System Science Data Center, National Science & Technology Infrastructure of China.^2^ Simultaneously, the percentage of scrub, farmland, grassland, forest and wetland area in each county of Sichuan Province in 2010 was manually extracted from the land cover dataset of the same data center. Furthermore, the altitude data of each county were obtained from the Global Change Research Data Publishing and Repository.^3^ Besides, to assess the contribution of the variables to ST occurrence, socioeconomic variables were also collected. In this study, the 1 km grid GDP datasets of Sichuan Province in 2019 were obtained from the Resource and Environmental Science and Data Center^4^ to describe economic differences. Moreover, a gridded urban accessibility dataset of approximately 1 km by 1 km that were designed to estimated travel time to the nearest city of 50,000 or more people were obtained from the Joint Research Centre of the European Commission^5^ to estimate the impact of trade and travel on disease transmission. The database of contributing variables of ST was established by corresponding the variables collected above with the maps of administrative divisions at county level. The base map comes from the National Catalog Service for Geographic Information.^6^

### 2.3. Analysis of epidemiological characteristics of ST

The Joinpoint regression model is mainly used to analyze the trend change characteristics of the time series data. Its dependent variables for applicable data mainly include the number of cases, incidence rate, or composition ratio, etc. It can be selected when the type of data distribution conforms to a normal, exponential or Poisson distribution (35). This method has been widely used since its introduction (25, 36). In this study, Joinpoint regression model was employed to examine the incidence trends from 2006 to 2021 and to calculate the annual percentage change. The annual incidence rates were calculated by dividing the number of ST cases by the gross population of a given year. The population data were obtained from the Sichuan Provincial Bureau of Statistics (37).

As a spatial statistical method, global spatial autocorrelation has been used to describe the relationship between study regions, measuring the degree of aggregation or dispersion (38, 39). In brief, if the Moran index is greater than 0, the distribution is spatially aggregated. If the Moran’s I index is less than 0, the distribution is spatially dispersing. If the Moran’s I Index is equal to 0, the distribution is spatially random. In this study, the global spatial autocorrelation method was used to investigate the spatial correlation of scrub typhus at county level in Sichuan Province each year.

Joinpoint version 4.9.1.0 was used to examine the incidence trends, ArcGIS version 10.3 was used for spatial analysis, data drawing and model output.

### 2.4. Assessment of the risk factors of ST occurrence

The boosting regression tree (BRT) model integrates the advantages of both regression tree model and boosting tree model. Through continuous self-learning and optimization, prediction models are built to evaluate the characteristics and outcomes of predicted unknown data (40, 41). Such models are increasingly being used to predict disease risk (32, 42). In this study, the BRT model was employed to assess the risk factors of ST occurrence. The database of contributing variables of ST were employed as the predictors for ST occurrence. The 64 counties that reported ST cases were defined as the case group, while the 119 counties that did not report ST cases were considered as the control group. The control group were randomly sampled for 300 times at a case–control ratio of 1:1 to construct 300 datasets together with the case group. For each dataset, training samples and verification samples accounted for 75 and 25%, respectively. We fitted a BRT model to each dataset and calculated the average contribution and response curves of each contributing variables and the average of the possible prevalence probability of ST in each county. The modeling process was performed using the “dismo” and “gbm” packages in the R statistical programming environment. The primary parameters were set as follows: tree complexity = 4, learning rate = 0.005, bag fraction = 0.75, the distribution type is “Bernoulli,” with others kept as default values (40). The area under the curve (AUC) was used to judge the accuracy and robustness of the model. R statistical software version 4.2.2 was used for model construction.

## 3. Results

### 3.1. Epidemiological features

**Figure 1:**
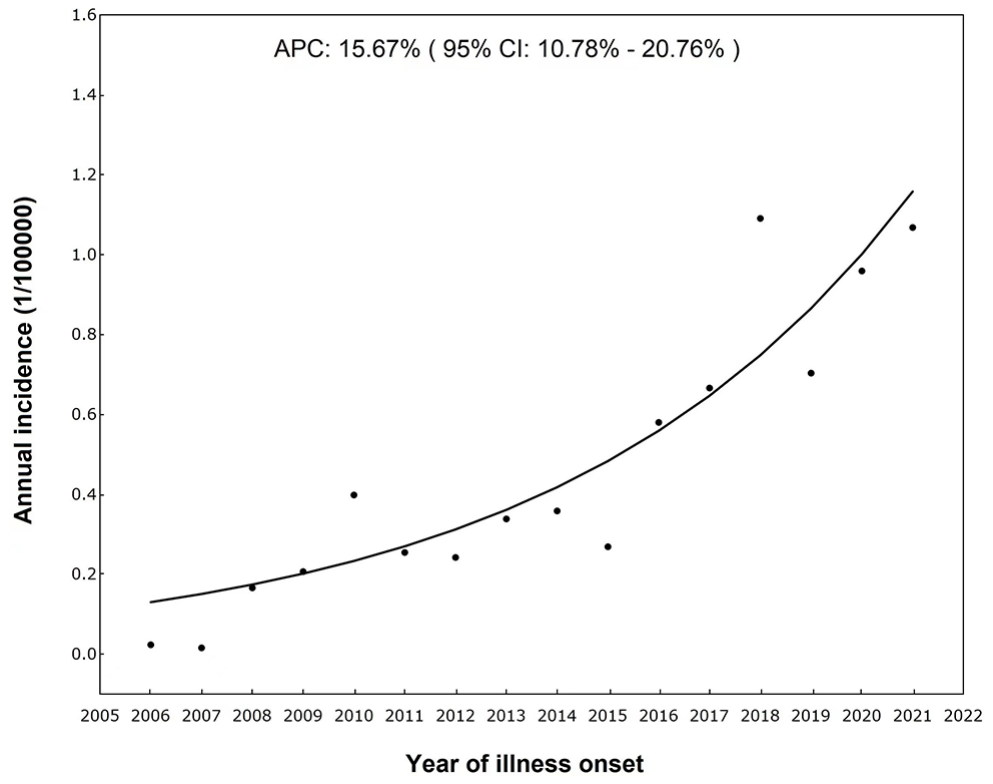
Temporal trends of incidence rates of ST in Sichuan (37).

It has been reported that there were 6,338 ST cases in Sichuan Province from 2006 to 2021, including 304 (4.80%) laboratory confirmed cases and 6,034 (95.20%) clinically diagnosed cases. The incidence rates increased over time, from 0.03 per 100,000 in 2006 to 1.12 per 100,000 in 2021 (Table 1). In accordance with the results of Joinpoint regression model, the annual percentage change in incidence was 15.67% (95% CI 10.78–20.76%, *p* < 0.001) (Figure 1).

**Table 1:** Demographic characteristics of ST cases in Sichuan (37).

Features	2006	2007	2008	2009	2010	2011	2012	2013	2014	2015	2016	2017	2018	2019	2020	2021	Total
No. confirmed cases	2	0	11	4	13	6	5	13	28	29	25	18	16	12	79	43	304
No. probable cases	20	15	131	178	331	207	204	277	283	207	472	557	918	592	744	898	6,034
Incidence (1/100,000)	0.03	0.02	0.17	0.22	0.42	0.26	0.26	0.36	0.38	0.29	0.61	0.70	1.13	0.72	0.98	1.12	0.48
**Sex**																	
Male	12	6	71	95	173	98	106	116	157	109	237	265	432	277	369	449	2,972
Female	10	9	71	87	171	115	103	174	154	127	260	310	502	327	454	492	3,366
**Age**																	
0–9	6	3	33	45	67	38	44	53	62	58	89	92	130	116	137	150	1,123
10–19	3	2	14	17	43	24	22	34	33	18	48	67	112	66	90	104	697
20–29	2	0	20	7	27	18	13	22	15	14	39	49	93	59	78	79	535
30–39	5	2	20	35	45	28	26	29	36	29	58	58	105	75	96	94	741
40–49	1	1	13	33	66	45	36	70	67	42	113	113	158	89	134	139	1,120
50–59	3	4	26	24	41	23	31	43	45	37	74	100	147	104	144	203	1,049
60–69	2	2	12	11	40	28	26	21	31	26	53	60	123	61	94	101	691
70–79	0	1	3	10	14	7	9	15	18	10	17	29	56	31	42	64	326
≥80	0	0	1	0	1	2	2	3	4	2	6	7	10	3	8	7	56
**Occupation**																	
Farmer	8	11	95	111	221	140	137	191	204	150	342	412	682	408	572	601	4,285
Scattered and preschool children	2	2	25	35	56	32	34	48	55	45	73	70	100	84	113	114	888
Student	6	2	19	24	49	28	30	35	36	25	54	73	123	89	104	127	824
Others	6	0	3	12	18	13	8	16	16	16	28	20	29	23	34	99	341
**Time from illness onset to diagnosis, d**																	
<3	2	6	31	45	82	55	45	51	56	47	123	173	234	150	199	228	1,527
3–7	13	5	66	85	160	87	92	128	150	124	243	197	367	247	283	332	2,579
≥7	7	4	45	52	102	71	72	111	105	65	131	205	333	207	341	381	2,232
No. in endemic county	13	5	14	19	22	17	13	19	19	14	23	24	32	35	34	43	64

The median age of the patients was 40 years (interquartile range [IQR], 15–54), increasing from 28 years (IQR, 9–41) in 2006 to 44 years (IQR, 17–56) in 2021. Among them, the median age of clinically diagnosed cases was 40 years (IQR, 13–55), and that of confirmed cases was 38 years (IQR, 13–55). Moreover, the 40–59 age group accounted for the largest number of cases (34.22%), The incidence rates were highest in 0–9 age group, followed by 50–59 age group, which were 0.77/100,000 and 0.63/100,000, respectively. During the study period, there were more cases reported in females. The ratio of male to female of the patients was 0.88, dropping from 1.2 in 2006 to 0.91 in 2021. Among them, this ratio of clinically diagnosed cases was 0.89, and that of confirmed cases was 0.83. Furthermore, the mean incidence rates were also higher in females (0.52/100,000) than in males (0.45/100,000). Among all ages, the incidence rates were higher in females vs. in males, except for the 0–9 and 10–19 age groups. The epidemiological surveillance of different occupations revealed that the number of cases was the largest in farmers, accounting for 67.61%. Among them, the proportion of farmers among clinically diagnosed cases and confirmed cases was 67.6 and 67.4%, respectively, both accounting for the highest percentage. Similar to the previous results of the age-specific incidence, the number of cases was also large among scattered and preschool children (14.01%) and students (13.00%). The majority of cases (75.91%) were diagnosed 3 days after the onset of illness, and approximately half of these cases were diagnosed 7 days after the onset of illness (Table 1).

### 3.2. Spatial and temporal distribution

**Figure 2:**
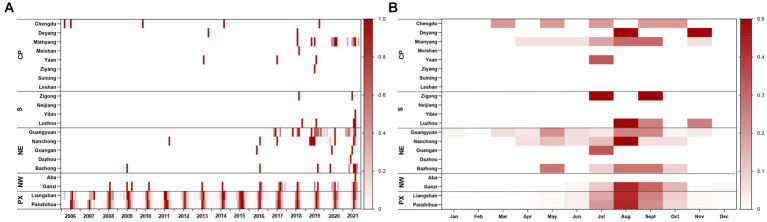
Heat map of ST for each city by region. **(A)** Time series of monthly cases during 2006–2021, standardized by the annual number of cases reported in each city and standardized to a range of 0–1. **(B)** Seasonal distribution of ST in each city, plotted as the average proportion of cases in each month from 2006 to 2021. CP, Chengdu Plain; S, Southern Sichuan; NE, Northeastern Sichuan; NW, Northwestern Sichuan; PX, Panxi region.

Most ST cases were distributed from June to October each year, peaking in August. A total of 95.38% of the cases occurred between June and October from 2006 to 2021. During the study period, almost all cases (97.27%) occurred in the Panxi Region, including Panzhihua and Liangshan Prefecture. The epidemic has slowly expanded to the northwest and northeast since 2015, and the number of affected counties increased from 13 in 2006 to 43 in 2021. Until 2021, a total of 19 cities (90.48%) and 64 counties (34.97%) had reported ST cases in Sichuan Province (Details were displayed in Figure 2). The global spatial autocorrelation analysis revealed that the incidence rates exhibited characteristics of clustering in each year, indicating a positive spatial correlation (Table 2).

**Table 2:** Global spatial autocorrelation analysis of ST in Sichuan (37).

Year	Moran’s I	Z-score	*p*-value
2006	0.48	12.24	<0.001
2007	0.08	7.00	<0.001
2008	0.32	9.21	<0.001
2009	0.09	7.14	<0.001
2010	0.25	6.57	<0.001
2011	0.25	8.14	<0.001
2012	0.26	8.47	<0.001
2013	0.21	9.42	<0.001
2014	0.32	9.17	<0.001
2015	0.21	6.02	<0.001
2016	0.26	6.99	<0.001
2017	0.19	5.07	<0.001
2018	0.31	8.28	<0.001
2019	0.30	7.81	<0.001
2020	0.30	7.71	<0.001
2021	0.27	6.97	<0.001

### 3.3. Risk assessment of the spatial distribution

**Figure 3:**
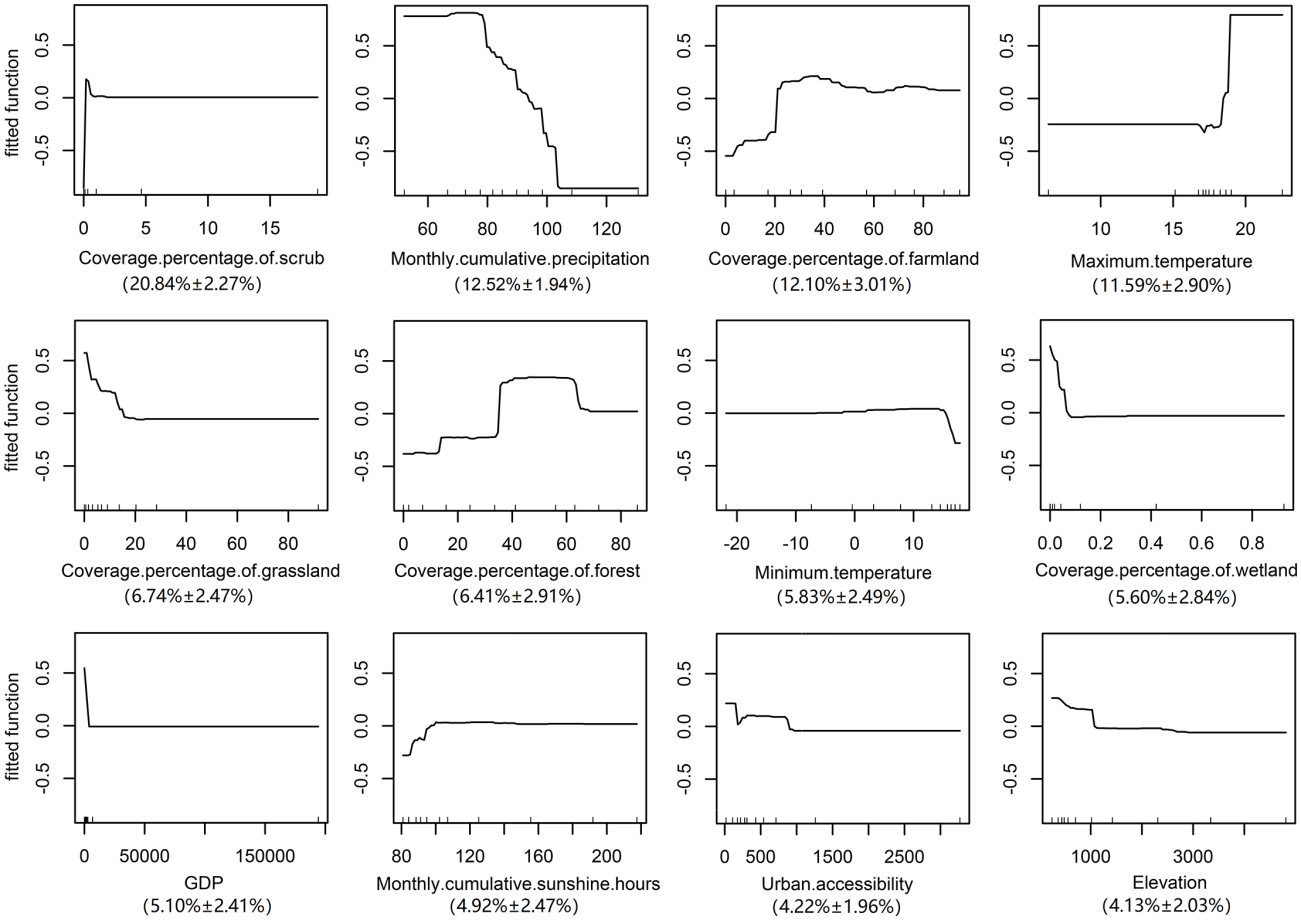
Response curves of each variable.

The validation statistics showed that The AUC value of the training data was 0.990 ± 0.011 s.e. and the validation data were 0.916 ± 0. 019s.e., suggesting that the BRT model in the current analysis can provide a good predictive accuracy. Variables including the scrub, precipitation, farmland and maximum temperature were considered as important predictors with a total contribution of 57.05% to the BRT model (Relative contribution for each variable: scrub, 20.84% ± 2.27%s.e.; precipitation, 12.52% ± 1.94%s.e.; farmland, 12.10% ± 3.01%s.e.; maximum temperature, 11.59% ± 2.90%s.e.) As the most contributing variable, the risk of ST increased sharply in areas with scrub cover, while there was no further increase in risk when the proportion of scrub exceeded 2%. Furthermore, the farmland and maximum temperature were both positively correlated with the risk of the disease, a rapid rise in the risk was noted when the area of farmland exceeded 20% or the temperature was higher than 17°C. Conversely, a negative correlation was detected between the precipitation and the risk of morbidity. When the precipitation was 70–80 mm, the risk of the disease was the highest. In addition, other variables that contributed to this model were grassland, forest, minimum temperature, wetland, and GDP (Relative contribution for each variable: grassland, 6.74 ± 2.47%s.e.; forest, 6.41% ± 2.91%s.e.; minimum temperature, 5.83% ± 2.49%s.e.; wetland, 5.60% ± 2.84%s.e.; GDP, 5.10% ± 2.41%s.e.). On the contrary, variables including sunshine, urban accessibility, and elevation did not contribute significantly to this model (Relative contribution for each variable: sunshine, 4.92% ± 2.47%s.e.; urban accessibility, 4.22% ± 1.96%s.e.; elevation, 4.13% ± 2.03%s.e.) (Details were displayed in Figure 3).

### 3.4. Infection risk areas

**Figure 4:**
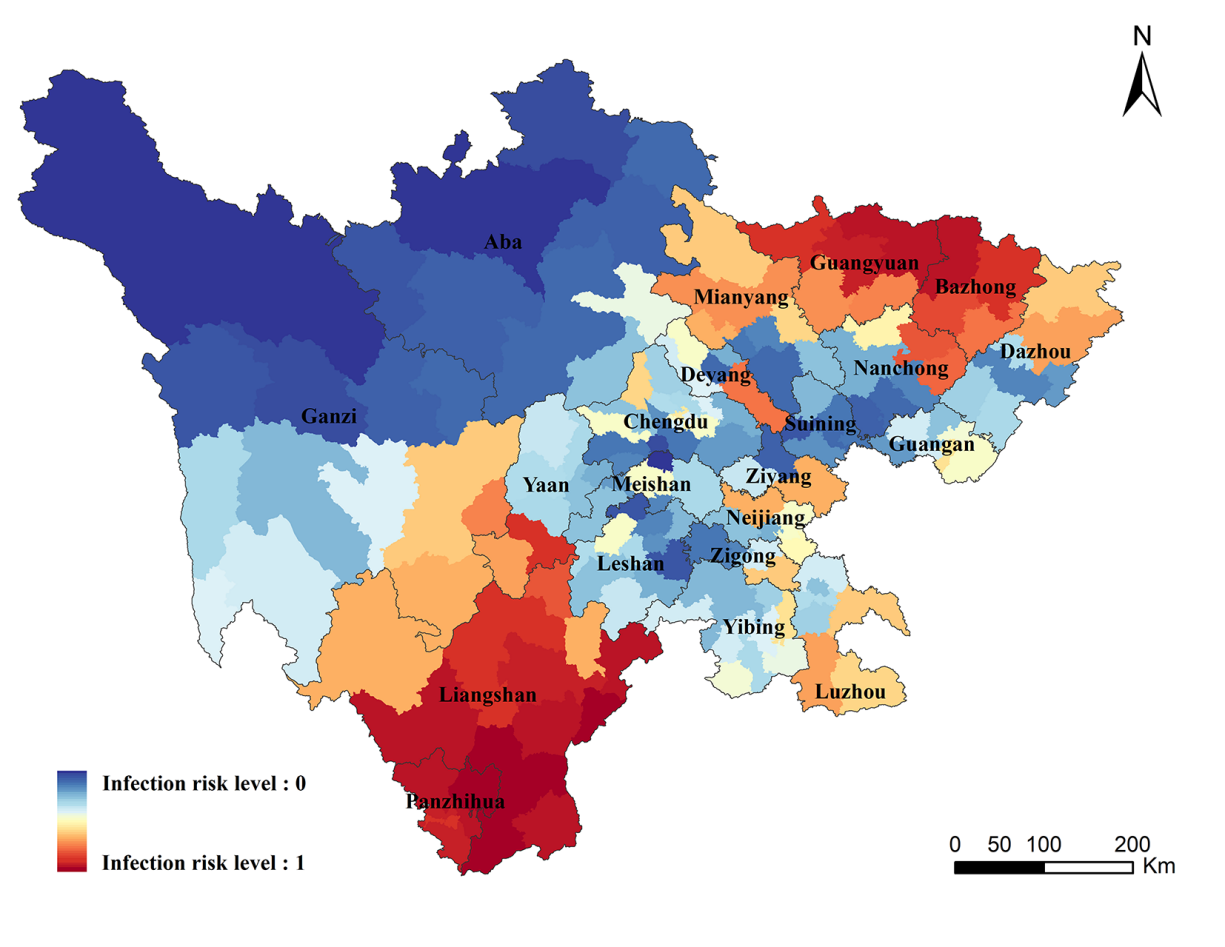
Geographic distribution of the predicted risk areas of ST in Sichuan Province.

Infection risk areas in Sichuan Province were mapped on the basis of the correlation between variables and ST in the BRT model. The areas with the highest risk of ST were concentrated in Liangshan, Panzhihua, Bazhong, and northern Guangyuan, of which Liangshan and Panzhihua were the traditional endemic areas. A small number of cases have been recorded in Bazhong and Guangyuan, however, a higher transmission risk was predicated in these zones. Moreover, our study found that some areas without case report detected a high risk of transmission, including Chengdu, Deyang, Mianyang, Ya ‘an in the central plain, and parts of Neijiang and Luzhou counties in southern Sichuan (Details were displayed in Figure 4).

The population size of the potential risk areas was computed by combining the predicated map with the population data of the seventh census. First, the areas with risk level above the threshold value of 0.5 were defined as the potential risk areas for ST occurrence. Then, the population data was overlaid on the potential risk areas to compute the population at risk. There were approximately 32.315 million people in the potential risk areas in Sichuan Province, accounting for 38.62% of the total population. All the people in Liangshan, Panzhihua, Bazhong and Guangyuan were at risk of transmission, and higher proportion of people living in Neijiang, Luzhou, Ziyang and Deyang were also at risk (Population proportion: 69.05% in Neijiang, 44.52% in Luzhou, 41.19% in Ziyang, 40.10% in Deyang).

## 4. Discussion

Based on the long-term surveillance data of ST in Sichuan Province, we comprehensively overviewed the epidemiological characteristics of the disease in Sichuan from 2006 to 2021. Then the BRT model was employed to identify variables that make sense and estimate the risk areas of ST occurrence.

**Figure 5:**
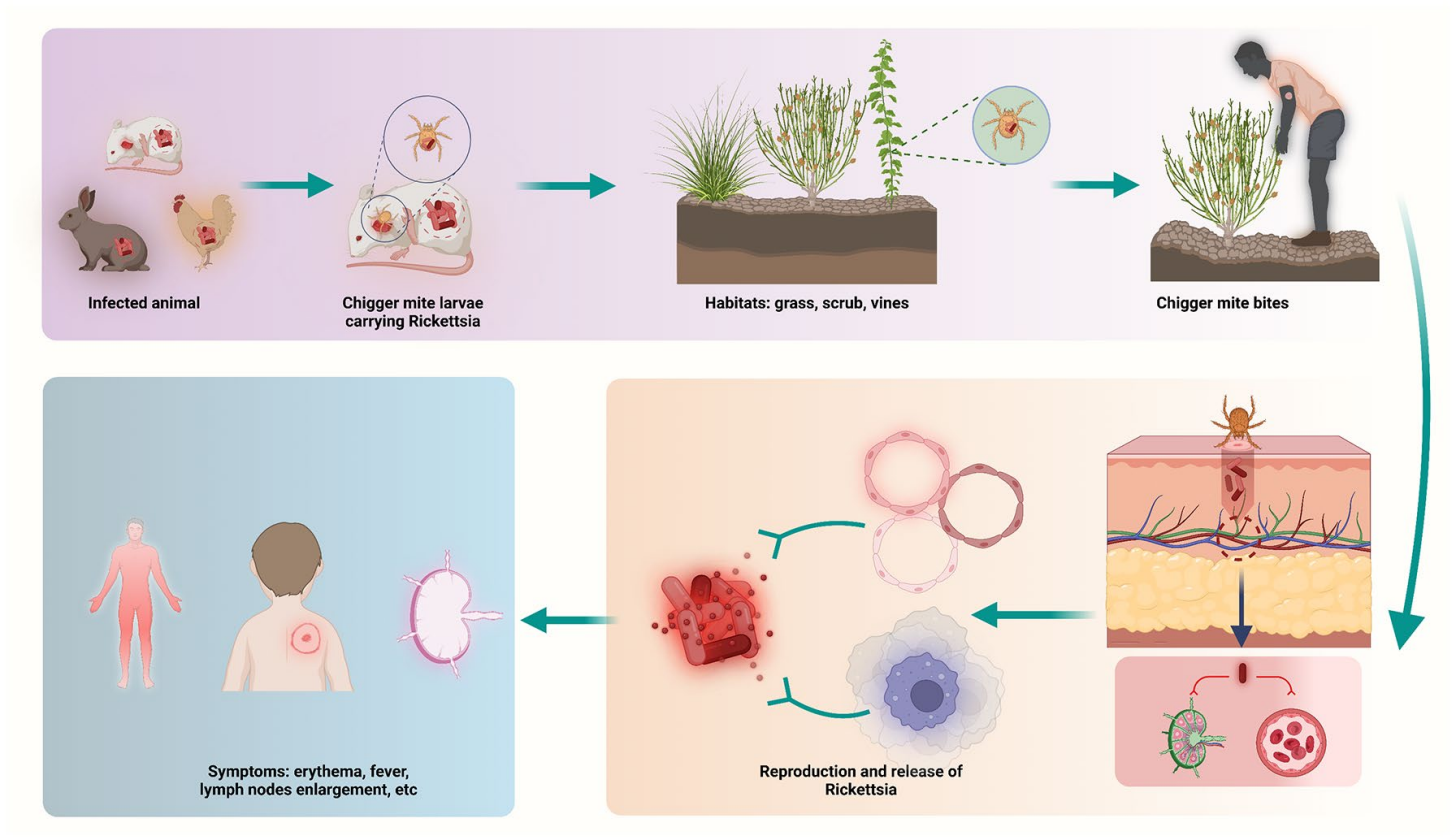
Schematic illustration on the process of ST infection.

ST is a mite-borne disease caused by *Orientia tsutsugamushi* (43). Generally, ST is transmitted by chigger (larval mites), which feed on rodents, such as rats, rabbits, and birds. The chiggers are both the vector and the natural reservoir. ST is spread to human through bites of infected chiggers. After infection, pathogens spread along the blood stream, invading vascular endothelial cells and macrophages (Figure 5). Similar to the symptoms and complications reported in other countries (44, 45), symptoms of ST usually appear within 10 days of being bitten, may include: fever, headache, muscle pain, a dark eschar at the bite site, enlarged lymph nodes, and rash (46, 47). Furthermore, complications usually involve the lungs, liver and cardiovascular system, causing dysfunction and even life-threatening in severe cases (48). For people at risk, prevention and control of ST is essential, and for infected patients, early diagnosis and treatment will determine the prognosis of the disease.

Since ST was listed as a reported disease by the National Notifiable Disease Reporting Information System in 2006, the reported incidence rates of ST in Sichuan Province have increased dramatically. In 2009, the Chinese Center for Disease Control and Prevention issued the technical guidelines for the prevention and control of this disease, and more cases were discovered. With the in-depth understanding of the disease, scholars gradually realized the impact of natural environment and socioeconomic variables on the spread and transmission of the disease, and managed to estimate the risk of disease through scientific methods and provide targeted prevention and control measures (25, 32, 42, 49, 50).

In this study, we noted that the incidence rates were relatively higher in preschoolers and boys were more susceptible than girls, which are supposed to be related to the fact that boys are more interested in outdoor play and sports. However, the majority of adult cases among adults were the older adult engaged in agriculture, and the incidence rates were higher in females. Commonly, youth and older adult male population in rural areas choose to work in cities for higher incomes, while elder women remain in the countryside, doing most of the agricultural work and taking care of the children. As everyone knows, the transmission of ST depends on the hosts and vectors. Staying in the grasslands and fields can increase the exposure to rodent hosts and chigger mites (32), leading to an increase in risk of ST. These findings are partially consistent with the study conducted by Yu et al. (31), who found that the number of ST cases in Jiangsu Province presented an inverted-U relation with age, moreover, the susceptible population of ST were farmers. Therefore, personal protection and health education to farmers and child guardians is recommended in risk areas to reduce the risk of infection.

The current study found that ST has obvious seasonality and clustering properties, and outbreaks are more likely to occur in the Panxi area in summer and autumn each year, which could be due to the differences in the distribution of chigger mites. According to the record, Panzhihua and Liangshan prefecture were the foci of ST. A variety of chigger mites were widely distributed in local areas, of which Leptotrombidium tsutsugamushi was the dominant species, and its activity peaked in the hot season ranging from June to September each year (51), which is consistent with our findings. Furthermore, the current study found that the risk areas increased rapidly and gradually spread to the economically developed urban plain areas, which may be associated with the growing demand for people to choose to go to the wild or parks for outdoor exercise. Another possible explanation is the increased number of the chigger mites and rodent hosts in cities, after all, it has been confirmed that both hosts and vectors can inhabit in urban environment (11). Taken together, it is recommended that necessary steps were supposed to be adopt to reduce the densities of rodents and chigger mites in endemic areas during the epidemic season, and personal protection and health education should be strengthened for travelers entering endemic areas.

The BRT model was employed to examine the correlation between variables and ST occurrence. The relative contribution of the scrub was observed to be high in the BRT model, suggesting the crucial role of land type in ST occurrence. Similar results were also reported by Hongwu Yao et al. (50), who found that scrub and grass land were the primary factors affecting the epidemic of scrub typhus in South natural foci in southern China. In general, scrub can be used as a platform for the activity of chigger mite larvae after hatching, and provide a suitable habitat for rodent hosts. In accordance with the land cover dataset, the scrub is primarily concentrated in Panxi area, which may partially explain the high incidence rates of ST in Panxi area. Moreover, previous research has demonstrated that the secondary vegetation types with changes in ecological environment provided ideal conditions for the survival and reproduction of tsutsugamushi (52). Hence, densely vegetated places near residences, villages, and ridges and ditches are considered to be the main places of tsutsugamushi infection (42). These are the regions we need to focus on to eliminate hosts and cut off transmission routes.

Previous study reported that ST occurrence is related to meteorological variables (34). The alterations of the meteorological variables such as temperature, precipitation and sunshine can not only alter chigger mite abundance, but also affect human behavior directly (35, 36). In the current analysis, a positive correlation was noted between maximum temperature and ST occurrence. Usually, rising temperatures provide a suitable living environment for hosts and vectors, and human activities increase as well. The main epidemic seasons for ST in Sichuan are summer and autumn (32). As temperatures rise, people spend more time in the field and their clothing becomes thinner, which increases the potential for human contact with chiggers or rats. In the current study, we noted that the Panxi region had the highest incidence of ST. The climate is primarily subtropical, with an average temperature above 20°C, which is suitable for the survival and reproduction of both host animals and chiggers. This is also relatively close to the prevailing suitable temperature proved by this study. In addition, a negative correlation was detected between precipitation and ST occurrence, which contradicts the understanding that humid environment is more conducive to chigger mite reproduction and thus increasing the risk of transmission. One possible explanation is that high humidity is detrimental to the life cycle of mites (50). In addition, several previous studies explained this phenomenon in terms of the interaction between human behavior and surrounding environment (32), namely, people were more inclined to be outside in the dry season vs. in the rainy season. Therefore, personal protection to reduce or avoid exposure to chigger mites is an effective measure for the prevention of ST.

There were more than 30 million people living in the potential risk areas in Sichuan Province. Especially those living in Liangshan, Panzhihua, Bazhong and Guangyuan. Moreover, some areas without case report detected a high risk of transmission, suggesting that surveillance in these places should be strengthened immediately.

Two limitations were noted in this research. First, our data come from a passive monitoring system, and there may be under-reporting and mis-reporting that affect the accuracy of the data. Second, we constructed our model without the use of vectors and hosts data, which are thought to be important variables affecting disease transmission. The next step is to collect more information on cases, pathogens, hosts, and vectors, including data on *O. tsutsugamushi* genotypes and the accurate distribution of Leptotrombidium mite species, and to conduct more detailed studies to formulate prevention and control.

In conclusion, we comprehensively evaluate the epidemiological characteristics of ST in Sichuan. For the first time, the BRT model was employed to investigate the underlying relationship of ST with meteorological, environmental and social variables in Sichuan Province. Many counties in Sichuan Province were estimated to be susceptible to ST. Our found can determine the priorities of disease control to achieve precise and effective allocation of resources.

## Data availability statement

The datasets presented in this study can be found in online repositories. The names of the repository/repositories and accession number(s) can be found below: The data of scrub typhus cases were available from China’s National Statutory Infections Disease Reporting Information System. Demographic data was obtained from the seventh population census of the National Bureau of Statistics of China (http://www.stats.gov.cn/). The meteorological data was obtained from the National Earth System Science Data Center, National Science and Technology Infrastructure of China (http://www.geodata.cn). the altitude data of each county were obtained from the Global Change Research Data Publishing and Repository (http://www.geodoi.ac.cn). The 1 km grid GDP datasets of Sichuan Province in 2019 were obtained from the Resource and Environmental Science and Data Center (https://www.resdc.cn). The gridded urban accessibility dataset was obtained from the Joint Research Centre of the European Commission (https://forobs.jrc.ec.europa.eu/products/gam/). The maps of administrative divisions at county level comes from the National Catalog Service for Geographic Information (https://www.webmap.cn/main.do?method=index).

## Author contributions

CY, RW, and YZ had the idea for the article. Data analysis was performed by YZ, MZ, and YQ. The first draft of the manuscript was written by YZ and MZ. YZ, MZ, YQ, LZ, DK, RW, and CY commented on previous versions of the manuscript. All authors contributed to the article and approved the submitted version.

## Funding

This study was supported by the National Institutes of Health (award no. 5RO1AI125842-02), Chongqing Science and Technology Program (cstc2020jscxcylhX0003), and Sichuan Science and Technology Program (2022YFS0641 and 2022YFS0052).

## Conflict of interest

The authors declare that the research was conducted in the absence of any commercial or financial relationships that could be construed as a potential conflict of interest.

## Publisher’s note

All claims expressed in this article are solely those of the authors and do not necessarily represent those of their affiliated organizations, or those of the publisher, the editors and the reviewers. Any product that may be evaluated in this article, or claim that may be made by its manufacturer, is not guaranteed or endorsed by the publisher.

## Footnotes

^1^ https://www.chinacdc.cn/tzgg/200901/t20090105_40316.htm

^2^ http://www.geodata.cn

^3^ http://www.geodoi.ac.cn/WebCn/doi.aspx?Id=887

^4^ https://www.resdc.cn/DOI/DOI.aspx?DOIID=33

^5^ https://forobs.jrc.ec.europa.eu/products/gam/

^6^ https://www.webmap.cn/main.do?method=index
