# Supplementary material for: Epidemiological analysis and risk prediction of scrub typhus from 2006 to 2021 in Sichuan, China
Source: Front Public Health. 2023 May 30;11:1177578. doi: 10.3389/fpubh.2023.1177578 (PMC10261982; doi:10.3389/fpubh.2023.1177578)
Supplement: Supplementary file 1 [file Table_1.DOCX]

Supplementary Material

Epidemiological Analysis and Risk Prediction of Scrub Typhus from 2006 to 2021 in Sichuan, China

**Yao Zhang1, Mengyuan Zhang1, Yao Qin 1, Lun Zhang 1, Dianju Kang1, Rongjie Wei 1, *, Changhong Yang 1, ***

*** Correspondence:** Rongjie Wei and Changhong Yang：[**changhong_yang@163.com**](mailto:changhong_yang@163.com)

**Supplementary Table**

**Supplementary Table. Diagnostic criteria of scrub typhus**

| Variable | Guidelines |
| --- | --- |
| Epidemiological history | 1.1 Field activities in endemic areas 1-3 weeks before illness onset, i.e., farming, fishing, camping, and straw collection. |
| Clinical manifestation | 2.1 Fever |
|  | 2.2 Lymphadenopathy |
|  | 2.3 Skin rash |
|  | 2.4 Specific eschars/ulcers |
| Laboratory tests | 3.1 An agglutination titer ≥1:160 in the Weil-Felix test using the OXK strain of Proteus mirabilis. |
|  | 3.2 A four-fold or greater rise in serum IgG antibody titers between acute and convalescent sera detected by using indirect immunofluorescence antibody assay (IFA). |
|  | 3.3 The detection of O. tsutsugamushi by polymerase chain reaction (PCR) in clinical specimens. |
|  | 3.4 The isolation of O. tsutsugamushi from clinical specimens. |
| Diagnosis and classification | Suspected case: |
|  | (1) a patient with item 1.1, 2.1, plus either 2.2 or 2.3, and was excluded from other diseases. or |
|  | (2) a patient with item 2.1, 2.2 and 2.3 during the local epidemic season of scrub typhus (May–November south of Yangtze River and October–November in north of Yangtze River). |
|  | Probable cases: |
|  | (1) a suspected case with item 2.4. or |
|  | (2) a patient with item of 1.1, 2.1, and 2.4. |
|  | Confirmed cases: |
|  | (1) a suspected case with any one of the items 3.2–3.4. or |
|  | (2) a probable case with any one of the items 3.1–3.4. |
